# A Surface Plasmon Resonance Plastic Optical Fiber Biosensor for the Detection of Pancreatic Amylase in Surgically-Placed Drain Effluent

**DOI:** 10.3390/s21103443

**Published:** 2021-05-15

**Authors:** Laura Pasquardini, Nunzio Cennamo, Giuseppe Malleo, Lia Vanzetti, Luigi Zeni, Deborah Bonamini, Roberto Salvia, Claudio Bassi, Alessandra Maria Bossi

**Affiliations:** 1 Indivenire srl, Via Alla Cascata 56/C, 38123 Trento, Italy; l.pasquardini@indiveni.re; 2 Department of Engineering, University of Campania “L. Vanvitelli”, Via Roma 29, 81031 Aversa, Italy; nunzio.cennamo@unicampania.it (N.C.); luigi.zeni@unicampania.it (L.Z.); 3 Unit of Pancreatic Surgery, University of Verona Hospital Trust, Department of Surgery, Dentistry, Gynecology and Pediatrics (DSCOMI), University of Verona, P. le Scuro 10, 37134 Verona, Italy; giuseppe.malleo@univr.it (G.M.); deborah.bonamini@univr.it (D.B.); roberto.salvia@univr.it (R.S.); claudio.bassi@univr.it (C.B.); 4 Fondazione Bruno Kessler-Sensors&Devices e Micro Nano Facility, Via Sommarive 18, 38123 Povo-Trento, Italy; vanzetti@fbk.eu; 5 Department of Biotechnology, University of Verona, Strada Le Grazie 15, 37134 Verona, Italy

**Keywords:** optical fiber sensors, surface plasmon resonance, plastic optical fibers, antibody, amylase, postoperative pancreatic fistula

## Abstract

Postoperative pancreatic fistula (POPF), the major driver of morbidity and mortality following pancreatectomy, is caused by an abnormal communication between the pancreatic ductal epithelium and another epithelial surface containing pancreas-derived, enzyme-rich fluid. There is a strong correlation between the amylase content in surgically-placed drains early in the postoperative course and the development of POPF. A simple and cheap method to determine the amylase content from the drain effluent has been eagerly advocated. Here, we developed an amylase optical biosensor, based on a surface plasmon resonance (SPR) plastic optical fiber (POF), metallized with a 60 nm layer of gold and interrogated with white light. The sensor was made specific by coupling it with an anti-amylase antibody. Each surface derivatization step was optimized and studied by XPS, contact angle, and fluorescence. The POF-biosensor was tested for its response to amylase in diluted drain effluents. The volume of sample required was 50 µL and the measurement time was 8 min. The POF-biosensor showed selectivity for amylase, a calibration curve log-linear in the range of 0.8–25.8 U/L and a limit of detection (LOD) of ~0.5 U/L. In preliminary tests, the POF-biosensor allowed for the measurement of the amylase content of diluted surgically-placed drain effluents with an accuracy of >92% with respect to the gold standard. The POF-biosensor allows for reliable measurement and could be implemented to allow for a rapid bedside assessment of amylase value in drains following pancreatectomy.

## 1. Introduction

Postoperative pancreatic fistula (POPF), the major cause of morbidity and mortality following pancreatectomy, is an abnormal communication between the pancreatic ductal epithelium and another epithelial surface containing amylase-rich fluid. From a mechanistic standpoint, a POPF is generated from a failure of the healing and sealing of a pancreatic-enteric anastomosis or from a direct parenchymal leak [1]. In 2005, an international panel of pancreatic surgeons agreed to establish an easy-to-apply clinical definition of POPF: an output from surgically or percutaneously-placed drains of any measurable volume of fluid on or after postoperative day 3, with an amylase content greater than 3 times the upper bound of normal serum amylase activity [1]. While a certain diagnosis of POPF can be established from postoperative day 3 and onwards, POPF is reliably predicted by measuring the amylase value from surgically-placed drains on postoperative day 1 [2]. This allows for tailored fast-track recovery pathways in low-risk patients and the implementation of mitigation strategies in high-risk patients [3]. Amylase level measurements (U/L) are typically based on a colorimetric test principle whose procedure takes about 30 min and is carried out in clinical laboratories by specialized personnel [4,5]. Having a fast, reliable, and easy to use point-of-care system to measure the levels of amylase in POPF would be of great importance for an early assessment of high-risk patients and for their treatment.

In general, the detection of amylases by means of biosensors are mostly based on electrochemical transduction systems and, in particular, are focused on the detection of amylases in saliva [6,7,8,9,10] and in human serum [11,12]. Concerning the measurement of amylase in drain fluids, to date just a single example of a biosensor is reported [13]. In this work, Förster resonance energy transfer (FRET) was selected as the working principle for the sensor and a labelled protein-assembly nanoprobe, sensitive to activated pancreatic proteases (i.e., elastase, α-chymostrypsin, trypsin), was able to optically detect activated pancreatic juices. In a preliminary test, the sensor was used to measure drain fluids, but the detection was solely of the drain fluid’s activated proteases. The results showed that the data of the FRET sensor positively correlated to the classification of the post-operatory conditions of the patients, which is routinary based on the determination of the amylase level. Nevertheless, the FRET-sensor did not strictly measure amylase. Given the key role played by the amylase levels in detecting POPF, we propose the development of a biosensor for fast, point-of-care, cost-effective and quantitative amylase measurements in surgically-placed drain effluent. The sensor is based on surface plasmon resonance (SPR) on a plastic optical fiber (POF) that is derivatized with a specific anti-amylase antibody. Previously, we reported on POF sensor systems with an innovative geometry [14,15] that were suitable for bio-applications [16]. The POF offers several advantages over glass fibers: it is easy to manipulate and, given its flexibility, it has a great numerical aperture and possesses a large diameter. The POF can be derivatized with a variety of recognition elements, such as proteins and antibodies [17], aptamers [18] and biomimetics, such as molecularly imprinted polymers [19], including molecularly imprinted nanogels [20], offering a versatile platform for biosensing. Moreover, the POF biosensor has demonstrated high sensitivity, down to the pM and fM levels, thus it matches the need for the detection of biomarkers in body fluids with low concentrations.

In the present work, we developed a SPR-POF-based biosensor for detection of amylase from the surgically-placed drain effluent of patients undergoing pancreatectomy. For this purpose, the POF surface was sputtered with a thin layer of gold (60 nm) that was chemically modified through the formation of a self-assembling monolayer (SAM) using α-lipoic acid, followed by covalent binding of the anti-amylase antibody to the surface by classical ethyleneimine-carbodiimide coupling chemistry. It was anticipated that the POF-sensor would be able to quantitatively detect amylase in PO fluids in a timespan of just a few minutes, offering quick response time for the assessment of the patient’s condition.

## 2. Materials and Methods

### 2.1. Substrates and Reagents

Gold substrates were prepared by depositing 10 nm of titanium on a silicon substrate (100), followed by 100 nm of gold purchased from MicroFabSolution srl (Trento, Italy).

Bovine serum albumin (BSA, A7030), α-lipoic acid (T5625), anti-α-amylase produced in rabbit fractionated antiserum (A8273) (IgG AMY), anti-mouse polyvalent immunoglobulins (G,A,M)−FITC, antibody produced in goat (F1010) (IgG FITC), and all of the powders for buffer solutions were purchased from Sigma-Aldrich srl (Milan, Italy). The mouse IgG1, kappa monoclonal [MOPC-21]—isotype control (ab18443) (IgG) was purchased from Abcam (Cambridge, U.K.). SuperSignal West Femto Chemiluminescent Substrate kit (34095), 1-ethyl-3-(3-dimethylaminopropyl) carbodiimide hydrochloride (EDC, 22980) and N-hydroxysulfosuccinimide (Sulfo-NHS, 24510), and albumin from bovine serum, tetramethylrhodamine conjugate (BSA-TAMRA, A23016), were purchased from Thermo Scientific (Rockford, IL, USA).

### 2.2. Horseradish Peroxidase (HRP) Conjugated Amylase

The protocol for the two-step coupling of proteins by means of EDC and sulfo-NHS was done according to [21]. Human amylase (Sigma, Milan Italy) was dissolved at 5 mg/mL in 10 mM of MES pH 5 buffer. The HRP was dissolved at 5 mg/mL in 100 mM MES pH 6. Sulfo-NHS was prepared at 200 mM in 10 mM of MES pH 5 buffer. EDC was prepared at 200 mM in 10 mM of MES pH 5 buffer. A quantity of 44 µmoles of HRP was admixed with EDC and sulfo-NHS and left to react at room temperature for 15 min. Then, 42 µmoles of amylase were added and the proteins were left to react for 2 h at room temperature in the dark. The HRP-amylase conjugate was dialyzed O/N at 4 °C against 3 L of 20 mM phosphate buffer (PB) with a pH of 7.5. An aliquot was diluted ten times in buffer and quantified by spectrophotometer. The conjugate was purified on a FPLC with a gel permeation column Superdex 200 10/300 GL on an AKTA prime separation system (GE, Sweden). The elution buffer was PB 50 mM pH 7.4 and the flow rate was 0.3 mL/min.

### 2.3. Biological Samples

Biological samples were obtained on postoperative 1 from surgically-placed trans-anastomotic stents (PankreaPlus, Peter Pflugbeil Gmbh, Germany) or intra-abdominal drains (Easy flow, Redax, Italy) of patients undergoing pancreatic resection at the Unit of General and Pancreatic Surgery in the University of Verona Hospital Trust. Trans-anastomotic stenting and drain placement in the proximity of a pancreatic-enteric anastomosis or pancreatic raw surface is a standard practice following pancreatic resection. Patients signed informed consent for utilization of biologic samples (PAD-R protocol, 1101CESC).

### 2.4. Gold Surface Functionalization

**Figure 1:**
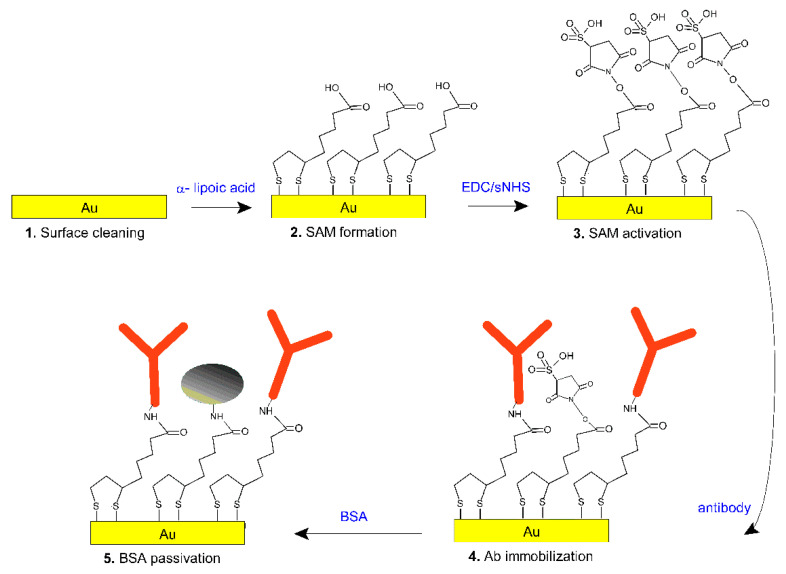
Scheme of the functionalization protocol: (**1**) gold cleaning with Argon plasma, (**2**) α-lipoic acid self-assembled monolayer, (**3**) activation with EDC/sulfo-NHS, (**4**) anti-amylase antibody immobilization and, finally, (**5**) surface passivation with bovine serum albumin (BSA).

The protocol is reported in Figure 1. Prior to functionalization, gold substrates were cleaned by argon plasma for 2 min at 6.8 W to remove organic contaminants. Then, substrates were immersed in α-lipoic acid ethanolic solution (8% in MilliQ water) at 0.3 mM for 18 h at room temperature. After incubation, three washings with MilliQ water were applied to remove excess solution. The activation of carboxylic groups was performed using a mixture of EDC and sulfo-NHS in 50 mM of MES buffer pH 5.5, spanning different molar ratios and incubation times to find the best conditions. Then, a specific antibody (Ig GAMY) or an aspecific one (IgG) were incubated in binding buffer (10 mM phosphate buffer, 138 mM NaCl, 2.7 mM KCl, pH 7.4) in a range between 1 ÷ 50 µg/sample for different times (20 ÷ 120 min). Finally, after three washings in binding buffer, surfaces were passivated by testing different BSA solutions (0.05 ÷ 1 mg/mL) for 30 min in binding buffer. Optimized conditions are reported in the Results and Discussion section in paragraph 3.

### 2.5. Surface Characterization

The functionalization process on the flat gold substrate was characterized using X-ray photoelectron spectroscopy (XPS), contact angle (CA), chemiluminescence and fluorescence measurements.

#### 2.5.1. XPS Measurement

A Kratos Axis Ultra DLD (Kratos Analytical Ltd., U.K.) instrument, equipped with a hemispherical analyzer and a monochromatic AlKα (1486.6eV) X-ray source in spectroscopy mode, was used to analyze the samples. The emission angle between the analyzer axis and the normal sample surface was 0° or 60° (sampling depth of approximately 10 or 2–3 nm [22]). The following core lines were acquired: O 1s, C 1s, N 1s, S 2p and Au 4f. The quantification, reported as a relative elemental percentage, was performed by using the integrated area of the fitted core lines (after Shirley background subtraction) and by correcting for the atomic sensitivity factors through a dedicated software [23]. This procedure provided a quantitative analysis, which was useful for the chemical characterization of the surface at different modification steps.

#### 2.5.2. Fluorescence Measurement

The FITC fluorescence signal on IgG and TAMRA on BSA molecules were monitored using a fluorescence microscope (Leica DMLA; Leica Microsystems, Germany) equipped with a mercury lamp and the fluorescence filter L5 and N2.1, respectively (Leica Microsystems, Germany). All samples were observed with a 20x magnification objective and measured with a cooled CCD camera (DFC420C, Leica Microsystems, Germany), analyzing the signal with Fiji software [24].

#### 2.5.3. Contact Angle (CA) Characterization

The static contact angle was measured using a home-made system, depositing 2 µL of deionized water droplets on the substrate (at least two drops per sample). The images were acquired with a CMOS camera and analyzed by Drop-Analysis, a plugin of Fiji software [24]. The results were reported as average value and standard deviations.

#### 2.5.4. Chemiluminescence Characterization

After incubation and washing, the samples were transferred into a black microplate. A 100 µL of chemiluminescent substrate was added to the wells and after 5 min the signal was acquired. The chemiluminescence signal of the HRP conjugated amylase was developed using the SuperSignal West Femto Chemiluminescent Substrate kit, according to manufacturer instructions, and measured through a Chemidoc MP Imaging System (BioRad) with 1 s of acquisition time. The measured signal was quantified using Fiji software [24].

### 2.6. Amylase Measurements on the POF-Biosensor

Sensing measurements on the POF-biosensor platform were performed by dropping ~50 μL of sample in binding buffer (10 mM phosphate buffer, 138 mM NaCl, 2.7 mM KCl, pH 7.4) over the sensing region; spectra were acquired over time for up to 8 min. After 8 min of incubation, the spectrum was acquired, then a washing step in the same buffer was applied and the spectrum was acquired again by dropping fresh buffer over the sensor. The following samples were used for the measurements: amylase denatured, effluent from surgically-placed intra-abdominal drains (*n* = 2) and pancreatic juice from intrapancreatic, trans-anastomotic stent (*n* = 1). All samples were diluted prior to measurement in binding buffer supplemented with Tween 20 0.02% v/v.

## 3. Results and Discussion

### 3.1. Optimization of the Surface Chemistry for the Preparation of the POF-Biosensor

The strategy adopted for the derivatization of the POF’s gold surface was adapted from [17]; the derivatization steps are schematized in Figure 1. The surface derivatization protocol was initially optimized on flat gold substrates and later transferred to the POF platforms.

**Figure 2:**
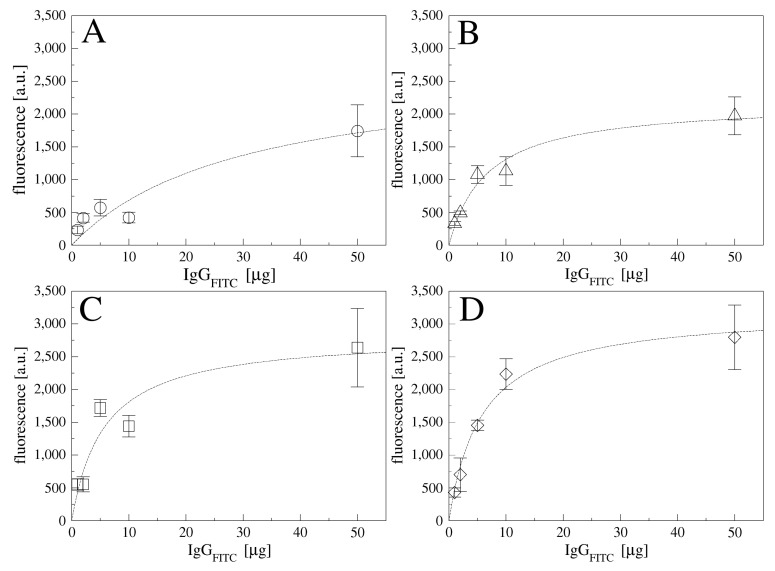
IgG FITC fluorescence on gold substrates functionalized at different concentration for different times: 20 min (**A**), 40 min (**B**), 60 min (**C**) and 120 min (**D**). Data are reported as mean value of five images acquired on the samples and error bars represent standard deviation.

The gold surfaces were coated with a self-assembled monolayer of α-lipoic acid according to [20]. Then, the effect of different molar ratios of EDC and sulfo-NHS (10:10, 20:10, 40:10, and 80:10 mM) on the immobilization of the antibody were tested as a function of the incubation time (20 ÷ 120 min), using a fluorescent antibody (IgGFITC; 5 g) as a probe and measuring the fluorescent signals by means of fluorescence microscopy. Figures S1 and S2 report the fluorescent (FITC) signal of IgG FITC derivatized surfaces and fluorescence images, respectively, for the different conditions tested. The mean fluorescence signal (Figure S1) suggested that each molar ratio tested guaranties an equal antibody immobilization up to 60 min. With increasing EDC concentrations, higher fluorescence signals were observed, mostly at 2 h of incubation, however some aggregates were imaged, as reported in Figure S2. Increasing the sulfo-NHS concentration did not report any advantages (data not shown). Based on the resulting images (Figure S2), a ratio of EDC-sulfo-NHS 10:10 mM for 40 min was selected for the further functionalization steps, given the uniform coverage achieved. The next step was to define the best concentration for the antibody to be immobilized and its incubation time over the EDC-sulfo-NHS activated surfaces. Using the fluorescent antibody IgG FITC in the range of concentrations between 1÷50 µg and for different times, we observed the binding curves over time as reported in Figure 2. The highest fluorescence signal was obtained by incubating 50 µg of IgG FITC for one hour, or 10 µg for two hours. Details on Langmuir fits are reported in Table S1. For further experiments, an incubation of 10 µg of antibody for two hours was selected.

A fundamental step for the preparation of a functional interface for the measurements of biological samples is the passivation of the surface. This step’s role is to prevent non-specific interactions between the surface and biomolecules of the sample, such as non-specific protein adsorption. Surface passivation is particularly important for optical biosensor applications because the non-specific binding events contribute to the optical signal, giving rise to false positives and to drift in the baseline.

**Figure 3:**
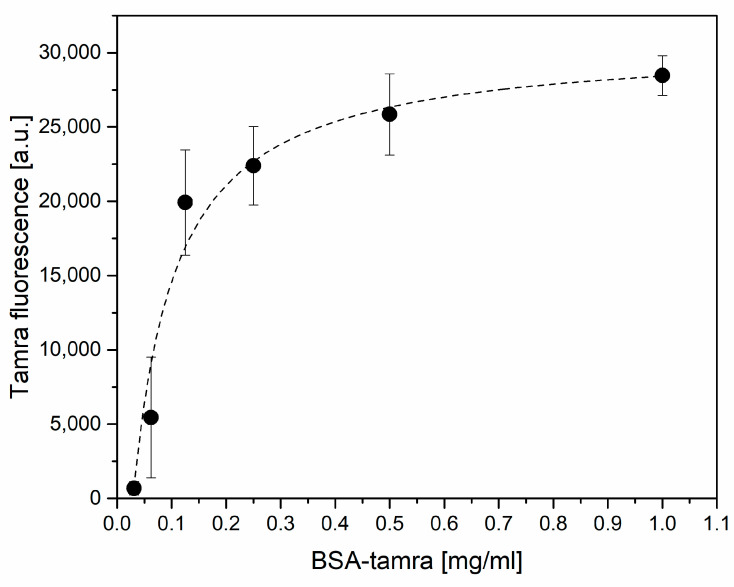
BSA-TAMRA adsorption to antibody functionalized surfaces after 30 min of incubation as a function of different BSA-TAMRA concentrations. Data are reported as the mean value of five images acquired on the samples and the error bars represent standard deviation.

The most common molecule used for passivation is bovine serum albumin (BSA). To set up the passivation step, we used a fluorescent, TAMRA-conjugated, BSA spanning a wide range of concentrations (0.03 ÷ 1 mg/mL in binding buffer), with each used to passivate the IgG functionalized gold surfaces for 30 min. The excess BSA was removed by washings in binding buffer and the surfaces were imaged with a fluorescent microscope. Figure 3 reports the fluorescence signal of TAMRA-BSA on the surface as a function of the TAMRA-BSA concentration. Data fitted with Langmuir showed a correlation coefficient of 0.96. A BSA concentration of 0.25 mg/mL was selected for the passivation step.

### 3.2. Physico-Chemical Characterization of the Anti-Amylase Flat Gold Surface

Each step of the preparation of the IgG surfaces was physico-chemically characterized. Surface characterization was performed by means of X-ray photoelectron spectroscopy (XPS) and contact angle (CA) measurements. As reported in Table 1, the chemical composition of the gold surface changed after each modification step. The formation of the α-lipoic acid SAM was clearly highlighted by the increase in the carbon content, the appearance of a sulphur signal and the decrease of the gold signal, confirming the coverage of the gold surface. The antibody immobilization was confirmed by the nitrogen appearance, the increase in oxygen and carbon content, and the decrease of the sulphur (typical of α-lipoic acid chains) and gold signal. A more detailed analysis was performed acquiring the core line levels for the different elements. Table S2 and Figure S3 report the quantification and the core lines at 60° take-off angle to highlight the differences between the samples. The α-lipoic acid immobilization (Au + SAM sample in Table 1, Table S2 and Figure S3) was confirmed by the appearance of a carboxylic moiety at around 289 eV and gold bound thiolate at around 162 eV in agreement with the literature [25]. After the antibody immobilization, the shape of the carbon core line greatly modified (Figure S3), increasing in the components related to C-O, C-N bonds at 286.5 eV and N = C-O bonds at 288.4 eV, in agreement with published works [26,27].

Contact angle measurements, reported in Table 1, confirmed the gold substrate modification after α-lipoic acid immobilization given the increase in hydrophobicity of the surface. The further derivatization with the antibody layer conferred a slightly more hydrophilic character to the surface.

### 3.3. Functional Characterization of the Anti-Amylase Flat Gold Surface

**Figure 4:**
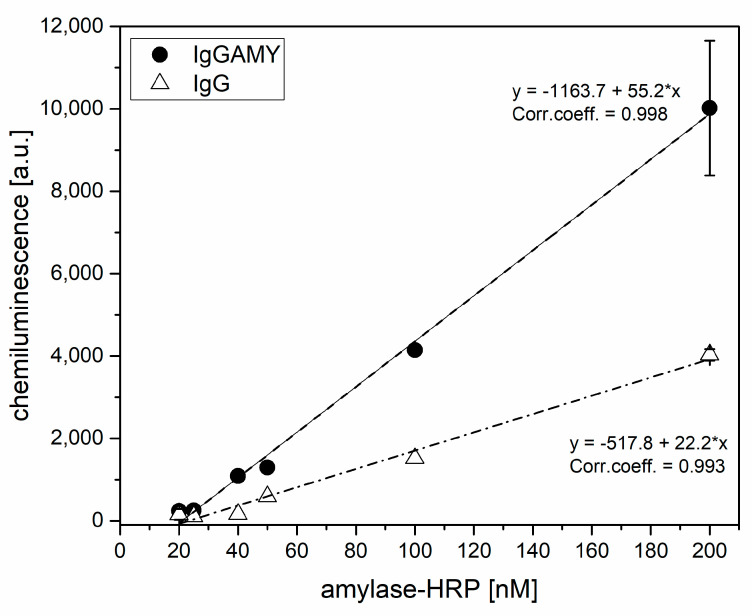
HRP-amylase chemiluminescence signal detected on flat gold surfaces prepared with specific antibody (IgG AMY) or aspecific one (IgG) in the linear range. Data are reported as the mean of two independent experiments and the error bars represent standard deviation.

Before proceeding with the preparation of the POF-biosensor, the selectivity of the anti-amylase (IgGAMY) flat gold surfaces for amylase was compared to that of non-specific IgG surfaces. The functional test was performed by assaying the surfaces for the binding of an HRP-conjugated amylase that was used as analyte. Increasing amounts of HRP-amylase were incubated onto both the IgG AMY and the IgG surfaces for 1 h, followed by three washings with binding buffer. To minimize the non-specific adsorption, the binding and washing buffers were supplemented with the neutral detergent Tween 20 at 0.02% v/v. The surfaces were then transferred into a black microplate and the chemiluminescent substrate was added. After 5 min, the signal was acquired with a 1 s exposure. Figure 4 shows the chemiluminescent signal for both IgG AMY and IgG surfaces as a function of the HRP-amylase concentration.

By referring the data of Figure 4 to the calibration curve, reported in Figure S4, the amount of HRP-amylase bound to the surface was estimated. Considering the incubation of 200 nM as the surface saturation, and by using the fitting parameters reported in Figure S4, a surface coverage of 16.7 pmol/cm^2^ was estimated. Figure 4 highlights the high non-specific adsorption to IgG antibody surface despite the addition of detergent in the incubation and washing steps. The specific antibody had a response 2.5 times higher than the aspecific one.

### 3.4. The POF-Biosensor Set Up and Its Use for the Detection of the Amylase in Surgically-Placed Drain Effluent

Finally, the POF-biosensor was prepared according to the optimized protocol, as defined in the Section 3.1, Section 3.2 and Section 3.3. The surface plasmon resonance (SPR) sensing probe was realized by modifying a multimode POF with a PMMA core of 980 μm and a fluorinated polymer cladding of 10 μm (a total diameter of 1mm), as described in [14]. In the first step, the cladding and part of the core of the POF were removed along half the circumference by a polishing process obtained by exploiting two different polishing papers (5 μm and 1 μm polishing papers). After this step, the interaction between the propagated light and the SPR phenomenon was improved by a buffer layer of Microposit S1813 photoresist spun (6000 rpm for 60 s) on the exposed core. The buffer layer presented a refractive index major then that of the POF core [14]. Finally, a thin gold film was sputtered onto the buffer layer. The obtained gold nano-film was 60 nm thick and presented a good adhesion to the substrate.

**Figure 5:**
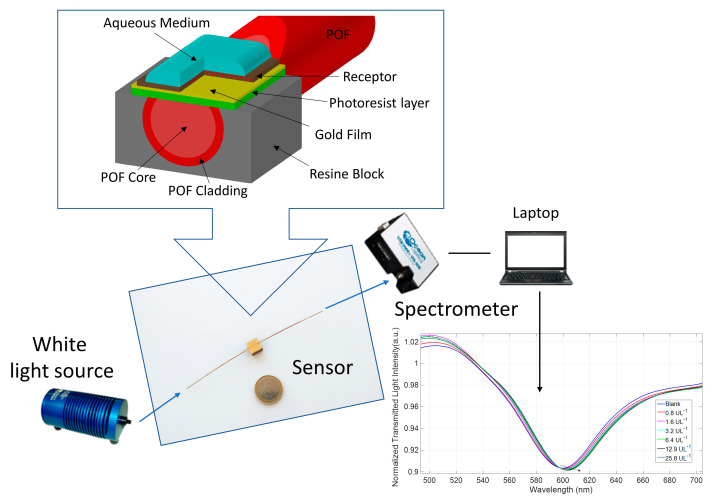
Setup of the POF-biosensor, as described in [14], with the equipment. The upper inset shows the cross section of the SPR-POF biosensor.

The POF-biosensor selectivity was obtained by covering the gold active surface with a very specific receptor layer for the considered analyte; in the present case, the anti-amylase antibody was used. The experimental measurements were carried out using a simple to use setup arranged to measure the transmitted light spectra using a halogen lamp, the SPR-POF sensor, and a spectrometer connected to a laptop. The halogen lamp exhibited a wavelength emission range from 360 nm to 1700 nm, while the spectrometer detection range was from 300 nm to 1100 nm. Figure 5 shows an outline of the sensor system with the equipment and a cross section of the SPR-POF sensor.

The SPR curves were obtained through Matlab software (MathWorks, Natick, MA, USA) by the experimental SPR-transmitted spectra, normalized to the spectrum acquired with air as the surrounding medium, for which the resonance condition was not satisfied (reference spectrum).

Before testing the ability of the POF-biosensor to detect amylase in the patient drain effluent, we estimated the theoretical binding capacity of the sensing surface to define the expected saturation of the sensor. Referring to the results reported in Section 3.3, we assumed that the IgG AMY covered the sensing surface at the density of 16.7 pmol/cm^2^. The POF-biosensor had a sensing area of 1 × 0.1 cm, which implied an expected maximal binding capacity of 1.67 pmol for the amylase. This corresponded to an expected maximal binding capacity of about 90 ng of amylase on the POF-biosensor.

Being that the intended final working environment for the POF-biosensor was the drain effluent of patients undergoing surgery, the sensor’s response and the calibration curve were directly studied on the surgically-placed drain effluent samples of known amylase contents. This choice allowed us to perform the measurements in the presence of a true matrix effect. In contrast, the use of pure human pancreatic amylase diluted in buffer seemed too dissimilar from the real samples for the present analytical purposes, such as the calibration curve on the POF-biosensor. Thus, pooled effluent with known content in amylase, as determined by standard laboratory measurement, were used.

**Figure 6:**
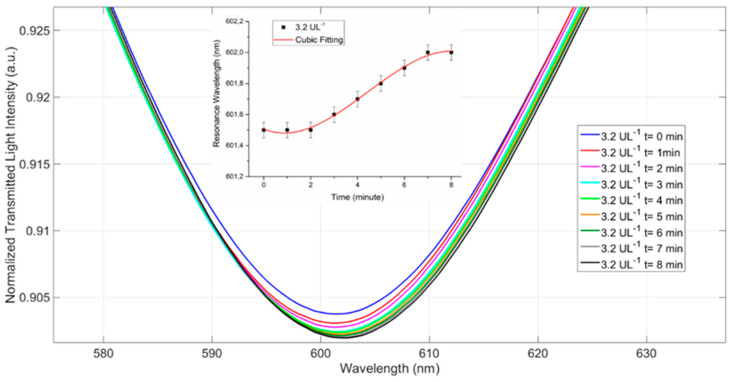
Kinetics of binding of 3.2 U/L amylase in surgically-placed drain effluent solution to the POF-biosensor. During the sample incubation (*t* = 8 min) the optical minimum shifts were recorded and plotted over time (inset). Eight minutes showed the endpoint of the kinetics and was chosen as the incubation time for all the measurements. The error bars were calculated on a same POF-biosensor platform and equal to 0.2 nm.

Initially, the kinetic response of the POF-biosensor for the binding of amylase was studied to define the optimal measurement time. As shown in Figure 6, following the deposition of 50 µL of sample (3.2 U/L amylase) on the POF-biosensor, the binding of the amylase to the sensor’s surface produced a shift in the optical minimum. The optical shifts of the sensor’s response were monitored over time and plotted (Figure 6, inset). Each experimental value was the average of three measurements obtained on the same POF-biosensor after the regeneration steps. The error bars reported in Figure 6 were calculated as the maximal measured standard deviation (equal to 0.2 nm). It was observed that after 7–8 min of incubation, the minimum stabilized. As a result, an incubation time of 8 min was set for all the following measurements.

**Figure 7:**
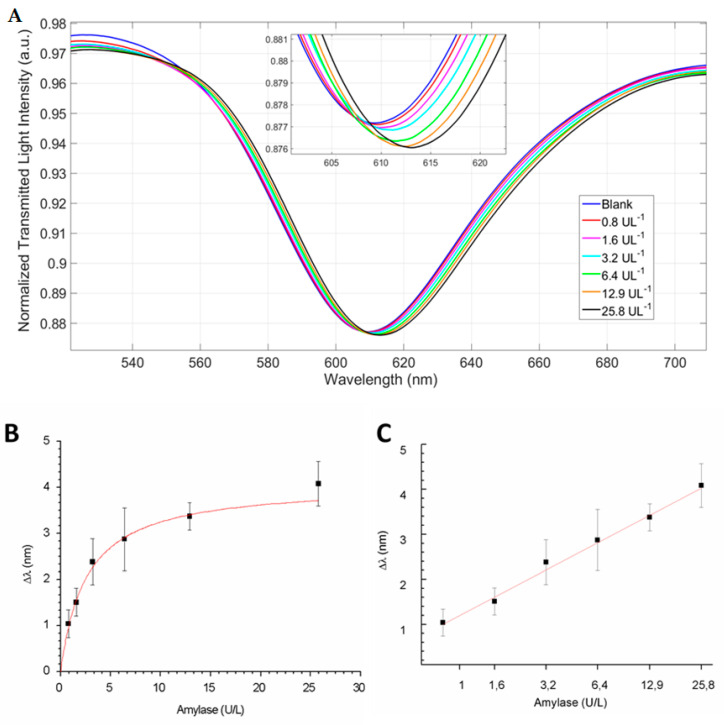
The (**A**) transmission spectra of SPR-POF platform functionalized with specific antibody (IgG AMY) and tested with amylase in surgically-placed drain effluent solution, (**B**) plasmon resonance wavelength variation (Δλ), with respect to the blank, versus the concentration of amylase content in drain effluent solution (U/L), and (**C**) linear fitting of data (mean value) obtained by two independent POF-biosensor batches. Error bars represent the standard deviation of measurements carried out on three different POF-biosensors prepared from three different batches.

The dynamic range of the response of the POF-biosensor for amylase was investigated. The drain effluent was diluted in a range of concentrations between 0.8 and 25.8 U/L and each sample was incubated for 8 min on the sensor surface. At the end of the incubation, the sensor was washed with PBS-Tween. A volume of 50 µL of PBS-Tween was dropped at the sensor surface and the signal was recorded. As shown in Figure 7A, increasing concentrations of amylase in the sample correlated to a red shift of the optical minimum. Figure 7B shows the binding isotherm, plotted as the Δλ of the optical minima as a function of the amylase units (U/L) and fitted with Langmuir equation: y = Bmax L/ (K + L), where Bmax was the maximal bound ligand, L was the ligand, i.e., the amylase herein expressed in U/L, and K was the half saturation. The apparent half saturation (K) for the POF-biosensor resulted at 1.989 U/L. The parameters for the Langmuir equation are reported in Table 2 and the chemical parameters for the POF-biosensor are shown in Table 3, where a limit of detection (LOD) roughly equal to 0.5 U/L is also reported. Moreover, the measurements were repeated on two sensing platforms, prepared from two different POF batches, and the mean data were linearly plotted using the mean values of Δλ min as a function of the logarithm of the concentration of amylase, expressed in U/L (Figure 7C). The linear equation was the following: y (Δλ)= 0.89 ln (x) + 1.41.

**Figure 8:**
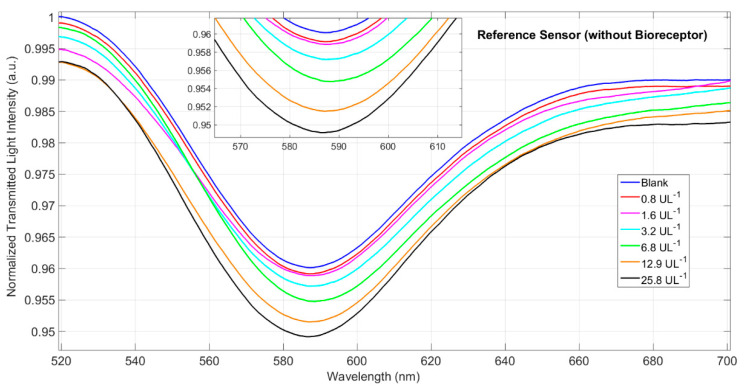
Transmission spectra of SPR-POF platform non-functionalized and tested with PO fluid solution.

To investigate the specificity of the POF-biosensor’s response to amylase, we used a passivated bare gold POF as a negative control. The optical response of the bare gold POF was monitored for dilutions of the drain effluent in the same range of concentrations used to draw the binding isotherm in Figure 7. The resulting optical minima on passivated gold POF for amylase 0.8–25.8 U/L are shown in Figure 8. Negligible optical shifts were observed, thus confirming the POF-biosensor had a specific response for amylase.

**Figure 9:**
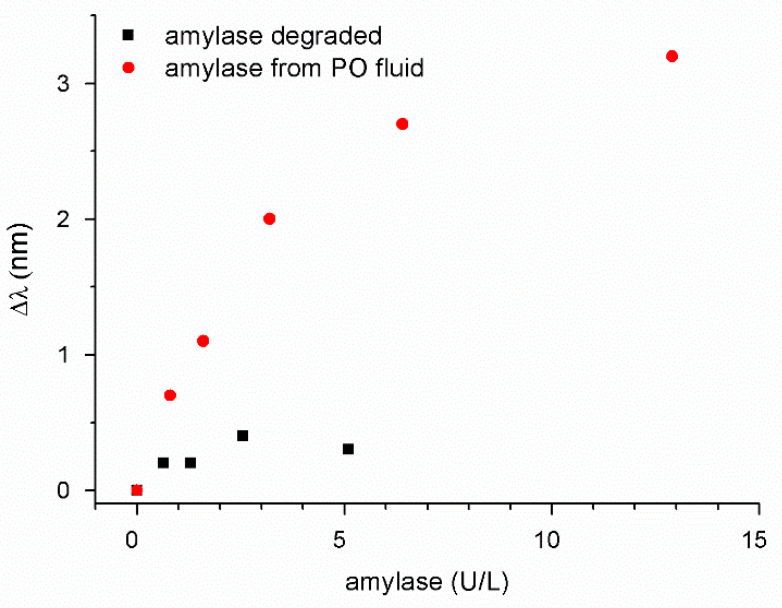
Plasmon resonance wavelength shifts (Δλ), with respect to the blank, versus the concentration of amylase (U/L). Red circles represent surgically-placed drain effluent; black squares represent amylase degraded (unfolded). Standard deviation associated with the measurements was 0.2 nm.

As a further control, we compared the POF-biosensor’s response for the drain effluent with the response of a degraded amylase. The solution of a commercial amylase in PBS was stored at room temperature for a few days to promote degradation and unfolding, then the amylase solution was diluted in the range 0.8–6 U/L and measured with the POF-biosensor. The results (Figure 9) clearly showed that the signal of the POF-biosensor for the degraded amylase result was negligible when compared with the non-degraded sample.

Lastly, we tested the POF-biosensor response blindfolded with a surgically-placed drain effluent of unknown amylase content. After dilution, the blind surgically-placed drain effluent was measured on the POF-biosensor and the U/L of the unknown sample was calculated using the linearized calibration curve (Figure 7C). The results shown in Table 4 demonstrate a good agreement between the POF-biosensor measurement and the amylase U/L determined with the enzymatic colorimetric test (IFCC), as also demonstrated by the calculation of the test accuracy, expressed in the Equation (1) according to [28]:

(1) $$100\%-\;│\frac{detected\;value-true\;value}{true\;value}\;\times \;100│\;$$

where *detected value* is obtained by POF biosensor and *true value* by IFCC test.

## 4. Conclusions

In this paper, we reported on the development of a SPR POF-biosensor selective for amylase and suitable for determining it in surgically-placed drain effluent following pancreatectomy. The SPR sensing surface was composed of a metallized D-shaped POF derivatized with a specific anti-amylase antibody. The results showed that the POF-biosensor measurement time was a few minutes, thus offering fast responses. The POF-biosensor was used to measure amylase directly on diluted drain effluent, thus reducing the analytical procedure to just a simple sample dilution. The POF-biosensor is the first described and based on the SPR principle. Currently, there are no sensors for the direct measurement of pancreatic amylase, thus the POF-biosensor is intended to fill the gap of fast and easy to use systems for the detection of the pancreatic amylase in patient’s postoperative conditions. Being that the POF-biosensor is suitable for use in remote sensing, it allows, in principle, performance of the analysis bedside instead of centralizing the collections of the results.

## Figures and Tables

**Table 1:** XPS characterization at 0° take-off angle and contact angle measurements on bare gold surface (Au), after α-lipoic acid coating (Au + SAM) and after antibody immobilization (Au + SAM + IgG AMY). XPS standard error does not exceed the 1–2% of the reported value.

	O 1s (%)	N 1s (%)	C 1s (%)	S 2p (%)	Au 4f (%)	CA [°]
**Au**	16.4	-	14.6	-	68.9	<5
**Au + SAM**	9.9	-	41.8	4.0	44.3	56.1 ± 3.4
**Au + SAM + IgG AMY**	18.9	7.0	48.2	2.9	22.9	52.6 ± 3.8

**Table 2:** Langmuir parameters of amylase detection in real solution by the SPR-POF biosensor.

Bmax		K		Statistics	
Value	Standard Error	Value	Standard Error	Reduced Chi-Sqr	Adj. R-Square
3.694	0.124	1.989	0.247	1.620	0.981

**Table 3:** Chemical parameters for amylase detection in real samples by SPR-POF biosensor.

	Parameters	Value
SPR-POF biosensor	K [ UL^−1^]	1.989
	Sensitivity at low *c* [nm/UL^−1^] (Sensitivity at low *c* = Bmax/K)	1.857
	LOD [UL^−1^] (3*standard deviation of blank/Sensitivity at low *c*)	0.48

**Table 4:** Comparison between the determination of the amylase (U/L) made by the POF-biosensor and the enzymatic colorimetric test used as the gold standard.

Sample	POF-Biosensor U/L	Enzymatic Colorimetric Assay U/L	Accuracy (%)
Drain effluent *n*.1	29,501 ± 6050	31,320	94.2
Drain effluent *n*.2	857 ± 76	794	92.1

## Supplementary Materials

The following are available online at https://www.mdpi.com/article/10.3390/s21103443/s1, Figure S1: IgGFITC fluorescence on gold substrates functionalized with α-lipoic acid and different molar ratio (mM) of EDC:sulfoNHS as a function of time. Data are reported as mean value of five images acquired on the samples and error bars represent standard deviation, Figure S2: Fluorescence images of IgGFITC antibody incubated at 5 μg/sample for different times (20 ÷ 120 min) on samples with different EDC:sulfoNHS molar ratio and acquired at 20x magnification objective. Scale bar is 100 μm., Figure S3: Main core line levels of different samples reported in Table S2 at 60° take-off angle, Figure S4: HRP-amylase calibration curve. Data are reported as mean value of two experiments and error bars represent standard deviation Table S1: Fitting parameters of the Langmuir curves y = A*x/(B + x) reported in Figure 2, Table S2: XPS characterization at 60° take-off angles on bare gold surface (Au), after α-lipoic acid coating (Au+SAM) and after antibody immobilization (Au+SAM+IgGAMY). XPS standard error does not exceed the 1–2% of the reported value.
