# Supplementary material for: A Surface Plasmon Resonance Plastic Optical Fiber Biosensor for the Detection of Pancreatic Amylase in Surgically-Placed Drain Effluent
Source: Sensors (Basel). 2021 May 15;21(10):3443. doi: 10.3390/s21103443 (PMC8156063; doi:10.3390/s21103443)
Supplement: Supplementary file 1 [file sensors-21-03443-s001.zip › sensors-1210120-supplementary.pdf]

Supporting Information

A Surface Plasmon Resonance Plastic Optical Fiber Biosensor for the Detection of Pancreatic Amylase in Postoperative Fluids.

Authors: Laura Pasquardini[1], Nunzio Cennamo[2], Giuseppe Malleo[3], Lia Vanzetti[4], Luigi Zeni[2], Deborah Bonamini[3], Roberto Salvia[3], Claudio Bassi[3], Alessandra Maria Bossi[5*]

[1] Indivenire srl, Via Alla Cascata 56/C, 38123 Trento, Italy;
[2] Department of Engineering, University of Campania "L. Vanvitelli", Via Roma 29, Aversa, Italy;
[3] Department of Surgery, Dentistry, Gynecology and Pediatrics (DSCOMI), University of Verona; Unit of Pancreatic Surgery, University of Verona Hospital Trust, P.Le Scuro 10, 37134Verona, Italy
[4] Fondazione Bruno Kessler-SD-MNF, Via Sommarive 18, 38123 Povo-Trento, Italy
[5] Department of Biotechnology, University of Verona, Cà Vignal 1, Strada Le Grazie 15, 37134 Verona, Italy

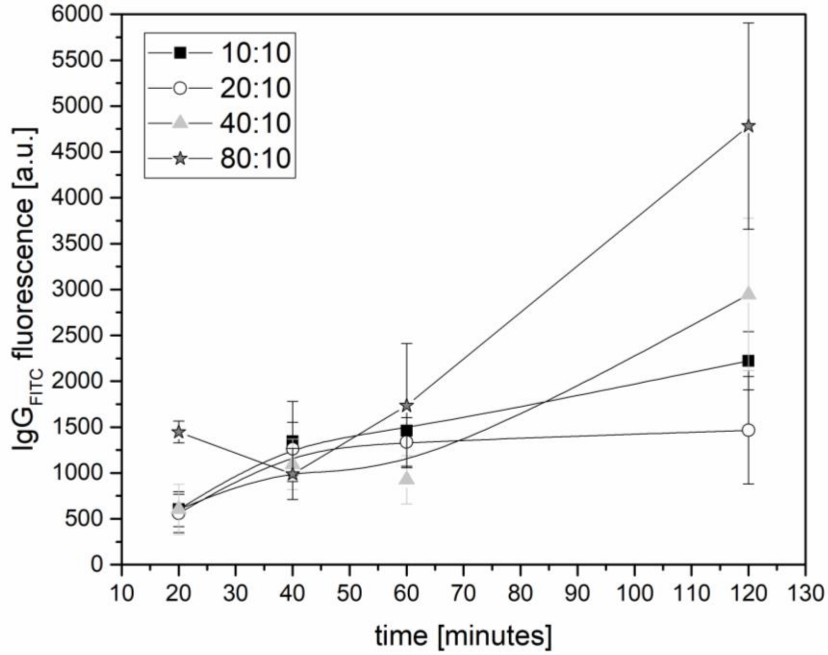

Figure S 1: IgG$_{FITC}$ fluorescence on gold substrates functionalized with α-lipoic acid and different molar ratio (mM) of EDC:sulfoNHS as a function of time. Data are reported as mean value of five images acquired on the samples and error bars represent standard deviation.

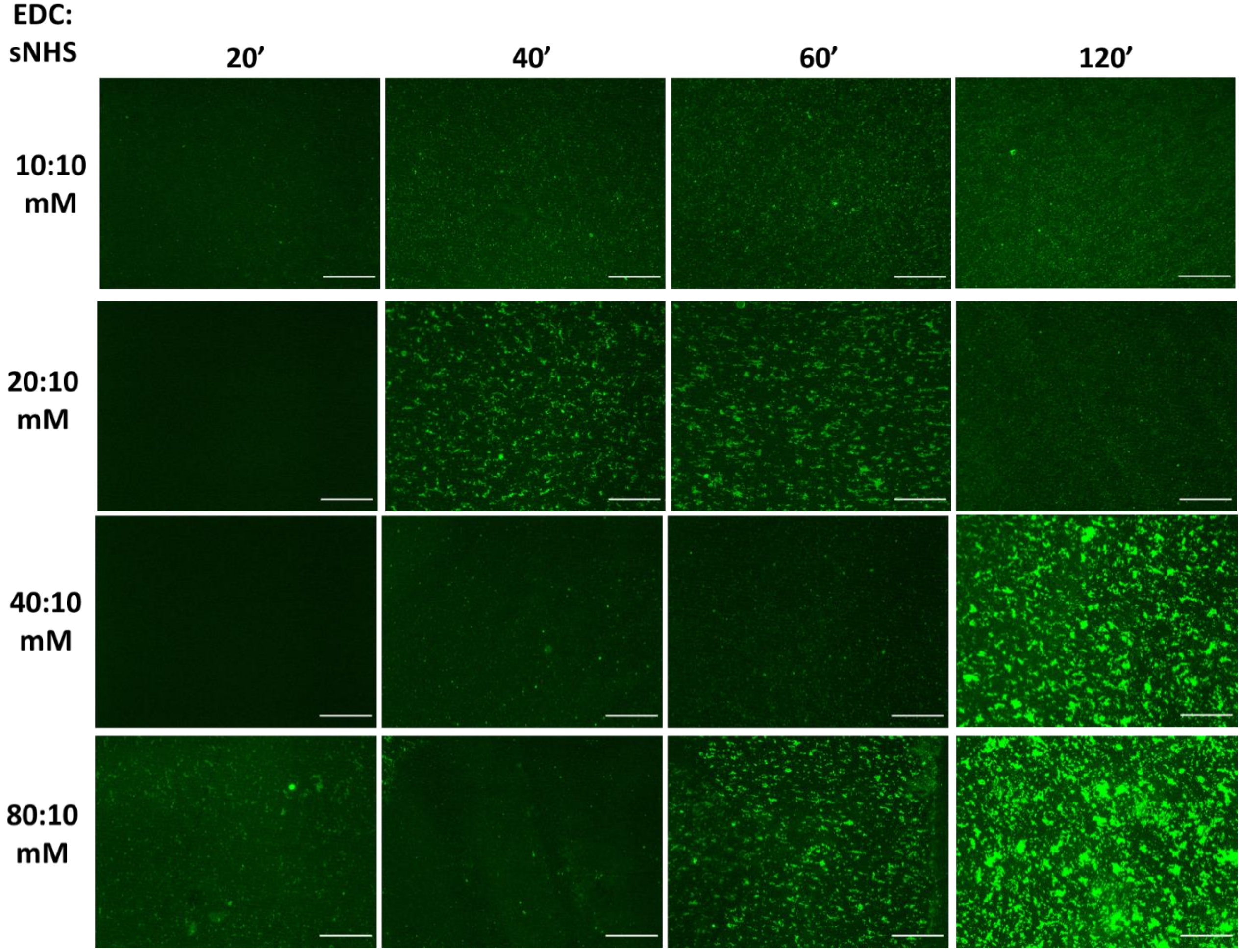

Figure S2: Fluorescence images of IgG_{FITC} antibody incubated at 5 μg/sample for different times (20 ÷ 120 minutes) on samples with different EDC:sulfoNHS molar ratio and acquired at 20X magnification objective. Scale bar is 100 μm.

*Table S1: Fitting parameters of the Langmuir curves y=A*x/(B+x) reported in Figure 2.*

Time [minutes]	A	B	Correlation coeff.
20	2738.31	29.92	0.95
40	2180.53	6.64	0.98
60	2831.44	5.65	0.94
120	3204.55	5.76	0.99

Table S2: XPS characterization at 60° take-off angles on bare gold surface (Au), after α-lipoic acid coating (Au+SAM) and after antibody immobilization (Au+SAM+IgG_{AMY}). XPS standard error does not exceed the 1–2% of the reported value.

	O 1s (%)	N 1s (%)	C 1s (%)	S 2p (%)	Au 4f (%)
**Au**	*15.3*	-	*32.1*	-	*52.6*
**Au+SAM**	*12.2*	-	*51.5*	*3.3*	*32.0*
**Au+SAM+IgG_{AMY}**	*18.3*	*7.8*	*57.5*	*2.0*	*14.3*

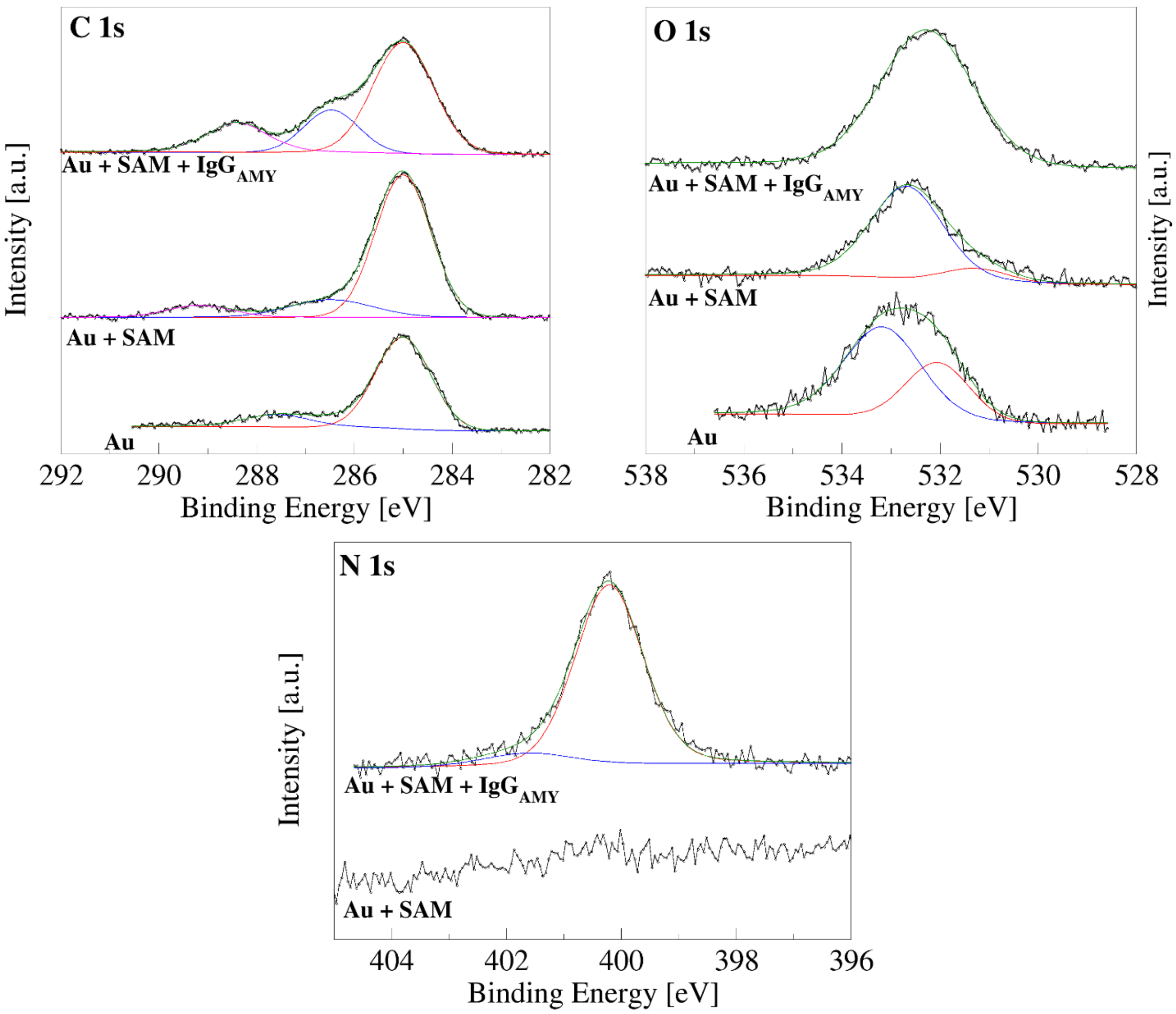

Figure S3: Main core line levels of different samples reported in Table S2 at 60° take-off angle.

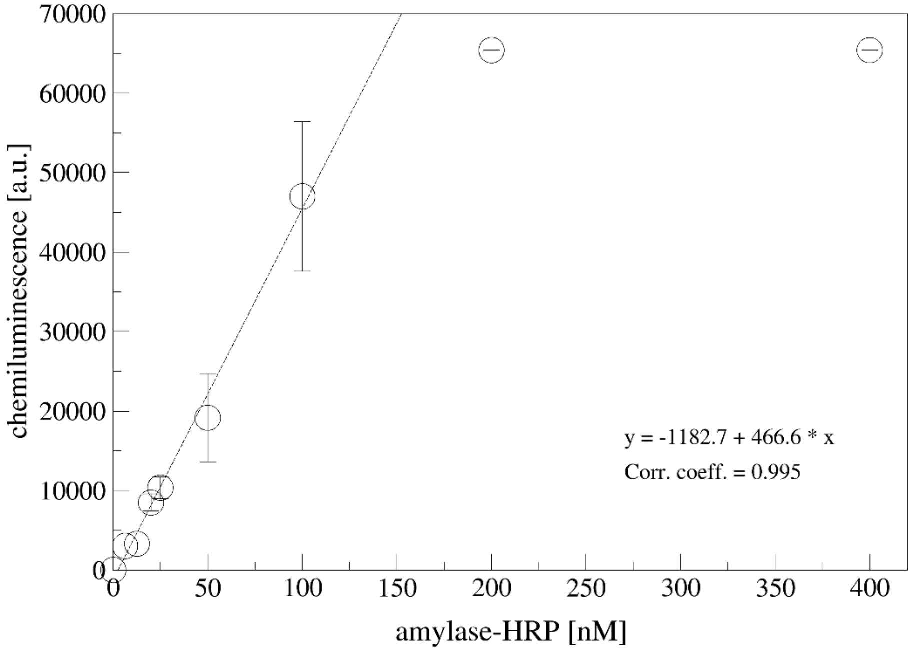

Figure S4: HRP-amylase calibration curve. Data are reported as mean value of four experiments and error bars represent standard deviation.
